# The influence of over-distraction on biomechanical response of cervical spine post anterior interbody fusion: a comprehensive finite element study

**DOI:** 10.3389/fbioe.2023.1217274

**Published:** 2023-08-15

**Authors:** Chih-Hsiu Cheng, Ping-Yeh Chiu, Hung-Bin Chen, Chi-Chien Niu, Mohammad Nikkhoo

**Affiliations:** ^1^ School of Physical Therapy and Graduate Institute of Rehabilitation Science, College of Medicine, Chang Gung University, Taoyuan, Taiwan; ^2^ Bone and Joint Research Center, Chang Gung Memorial Hospital, Linkou, Taiwan; ^3^ Department of Orthopedic Surgery, Chang Gung Memorial Hospital, Linkou, Taiwan; ^4^ Department of Biomedical Engineering, Science and Research Branch, Islamic Azad University, Tehran, Iran

**Keywords:** cervical spine biomechanics, poroelastic finite element modeling, personalized modeling, cervical anterior fusion, interbody cage, over-distraction

## Abstract

**Introduction:** Anterior cervical discectomy and fusion (ACDF) has been considered as the gold standard surgical treatment for cervical degenerative pathologies. Some surgeons tend to use larger-sized interbody cages during ACDF to restore the index intervertebral disc height, hence, this study evaluated the effect of larger-sized interbody cages on the cervical spine with ACDF under both static and cyclic loading.

**Method:** Twenty pre-operative personalized poro-hyperelastic finite element (FE) models were developed. ACDF post-operative models were then constructed and four clinical scenarios (i.e., 1) No-distraction; 2) 1 mm distraction; 3) 2 mm distraction; and 4) 3 mm distraction) were predicted for each patient. The biomechanical responses at adjacent spinal levels were studied subject to static and cyclic loading. Non-parametric Friedman statistical comparative tests were performed and the *p* values less than 0.05 were reflected as significant.

**Results:** The calculated intersegmental range of motion (ROM) and intradiscal pressure (IDP) from 20 pre-operative FE models were within the overall ranges compared to the available data from literature. Under static loading, greater ROM, IDP, facet joint force (FJF) values were detected post ACDF, as compared with pre-op. Over-distraction induced significantly higher IDP and FJF in both upper and lower adjacent levels in extension. Higher annulus fibrosus stress and strain values, and increased disc height and fluid loss at the adjacent levels were observed in ACDF group which significantly increased for over-distraction groups.

**Discussion:** it was concluded that using larger-sized interbody cages (the height of ≥2 mm of the index disc height) can result in remarkable variations in biomechanical responses of adjacent levels, which may indicate as risk factor for adjacent segment disease. The results of this comprehensive FE investigation using personalized modeling technique highlight the importance of selecting the appropriate height of interbody cage in ACDF surgery.

## 1 Introduction

Cervical degenerative pathologies including intervertebral disc (IVD) herniation and spondylosis remain the most common causes of cervical radicular pain (Zagra et al., 2013). Anterior cervical discectomy and fusion (ACDF) is widely acknowledged as the gold standard and has been consistently cited as one of the most effective and safe surgical interventions in cases where conservative management techniques prove ineffective (Robinson, 1955; Jacobs et al., 2011; Zagra et al., 2013). This procedure has garnered substantial recognition for its successful outcomes in relieving symptoms and addressing various cervical spine pathologies, such as disc herniation, spinal stenosis, and degenerative disc disease (Peng et al., 2022). The remarkable efficacy and safety profile of ACDF have contributed to its widespread adoption by clinicians and have made it a preferred option for patients who require surgical intervention to alleviate cervical spine-related conditions (Tsalimas et al., 2022). The ACDF surgical technique generally includes complete removal of the IVD and adjacent endplates (EPs), and replacement with autologous bone grafts or interbody cages (Song and Choi, 2014). The use of interbody cages has since evolved to become more popular in the last decade due to preserving the IVD height and cervical lordotic angle and reducing the operation time (Song and Choi, 2014; Jain et al., 2016). Despite significant advancements in interbody cage materials and structural designs over the past two decades, surgeons continue to face challenges in selecting the appropriate size for interbody cages (Kwon et al., 2005; Chong et al., 2015). The task of determining the correct size remains intricate and requires careful consideration of individual patient factors and specific surgical requirements (Hah et al., 2020; Lawless et al., 2022). Despite the progress in cage technology, ensuring the optimal fit and function of the interbody cage remains a critical aspect of successful surgical outcomes for patients. Some surgeons tend to select larger-sized interbody cages during ACDF to restore the index IVD height. Nonetheless, larger-sized interbody cages may alter the biomechanical response of adjacent levels post-surgery (Igarashi et al., 2019; Huang et al., 2021).

The clinical follow-up studies showed that increased stiffness at the ACDF level may cause adjacent segment disease (ASD) which is one of the most important concerns with a reported annual incidence of 2.9% (Hilibrand et al., 1999; Litrico et al., 2014; Kim et al., 2017; Goyal et al., 2019). Pseudoarthrosis, limited intersegmental mobility, and interbody cage subsidence are potential drawbacks associated with ACDF, emphasizing the essential requirement for meticulous surgical pre-planning (Shriver et al., 2015; Igarashi et al., 2019). Selecting a cervical interbody cage with appropriate height could be one of the key steps in ACDF, and may have critical influence on clinical outcome and the complication risk factors (Chong et al., 2015; Kong et al., 2016). In ACDF procedures, the process of distraction holds immense significance as it facilitates the separation of vertebral bodies, leading to neural structure decompression and supporting fusion (Kirzner et al., 2018; Lawless et al., 2022). Surgeons aim to achieve an optimal intervertebral height to ensure effective fusion and decompression (Hah et al., 2020). However, determining the precise threshold for what qualifies as “over-distraction” can vary substantially due to a multitude of factors. These factors encompass the patient’s individual anatomy, underlying medical condition, and the surgeon’s experience and skill level (Olsewski et al., 1994; Kirzner et al., 2018; Lawless et al., 2022). Induced modifications regarding ACDF surgery alter the biomechanical response of lower-cervical spine both in terms of motion patterns (i.e., kinematics) and load sharing (i.e., kinetics) (Wang et al., 2016; Huang et al., 2021). Despite the extensive number of clinical studies and *in-vitro* and *in-vivo* animal experiments conducted to evaluate the biomechanical performance of cervical interbody cages in ACDF, a controversy persists regarding the identification of potential risk and protective factors in ASD (Kwon et al., 2005; Chong et al., 2015; Huang et al., 2021). Although the wealth of research, a definitive consensus has not been reached, and different studies may yield conflicting findings (Li et al., 2020; Wong et al., 2020). The complexity of ASD and the multitude of factors that can contribute to its development make it challenging to pinpoint specific risk or protective factors definitively. As a result, ongoing research and comprehensive investigations are necessary to shed further light on this matter and potentially resolve the existing controversy.

While the achieved results from experimental and clinical studies provide valuable observations, nonetheless the detailed kinematics and kinetics of the cervical spine post-surgery are missing (Huang et al., 2021). Various FE models were developed to investigate the cervical spine with ACDF (Huang et al., 2021), based on which the effect of the interbody cage fusion on ASD can be explored (Zhang et al., 2016; Li et al., 2020; Khalaf and Nikkhoo, 2022). In addition to the comparative FE studies consequent to ACDF and dynamic systems (i.e., arthroplasty implants) (Choi et al., 2021; Khalaf and Nikkhoo, 2022), several investigations focused on evaluating the number of fused levels (Lopez-Espina et al., 2006; Hua et al., 2020), the materials and design of interbody cages (Chong et al., 2015; Zhang et al., 2016; Liu et al., 2017; Li et al., 2020) on the incidence of ASD. However, most of related FE simulations in literature were performed based on one geometry or very limited number of geometries (Kim et al., 2018; Huang et al., 2021). This limitation can introduce uncertainty in comparative studies and may have adverse effects on their consistency and predictive ability in clinical applications (Laville et al., 2009; Nikkhoo et al., 2019). Therefore, to fill the gap of knowledge, a comprehensive FE study to enhance the understanding of over-distraction during ACDF surgery on the biomechanical response of adjacent levels could be beneficial for clinicians. The use of geometrically personalized FE simulations may well improve the results and augment the prediction capability. Hence, this study utilized a personalized poroelastic FE modeling technique to evaluate the effect of larger-sized interbody cages on the cervical spine with ACDF under both static and cyclic loading.

## 2 Materials and methods

### 2.1 Development of the personalized lower-cervical spine FE models

**FIGURE 1:**
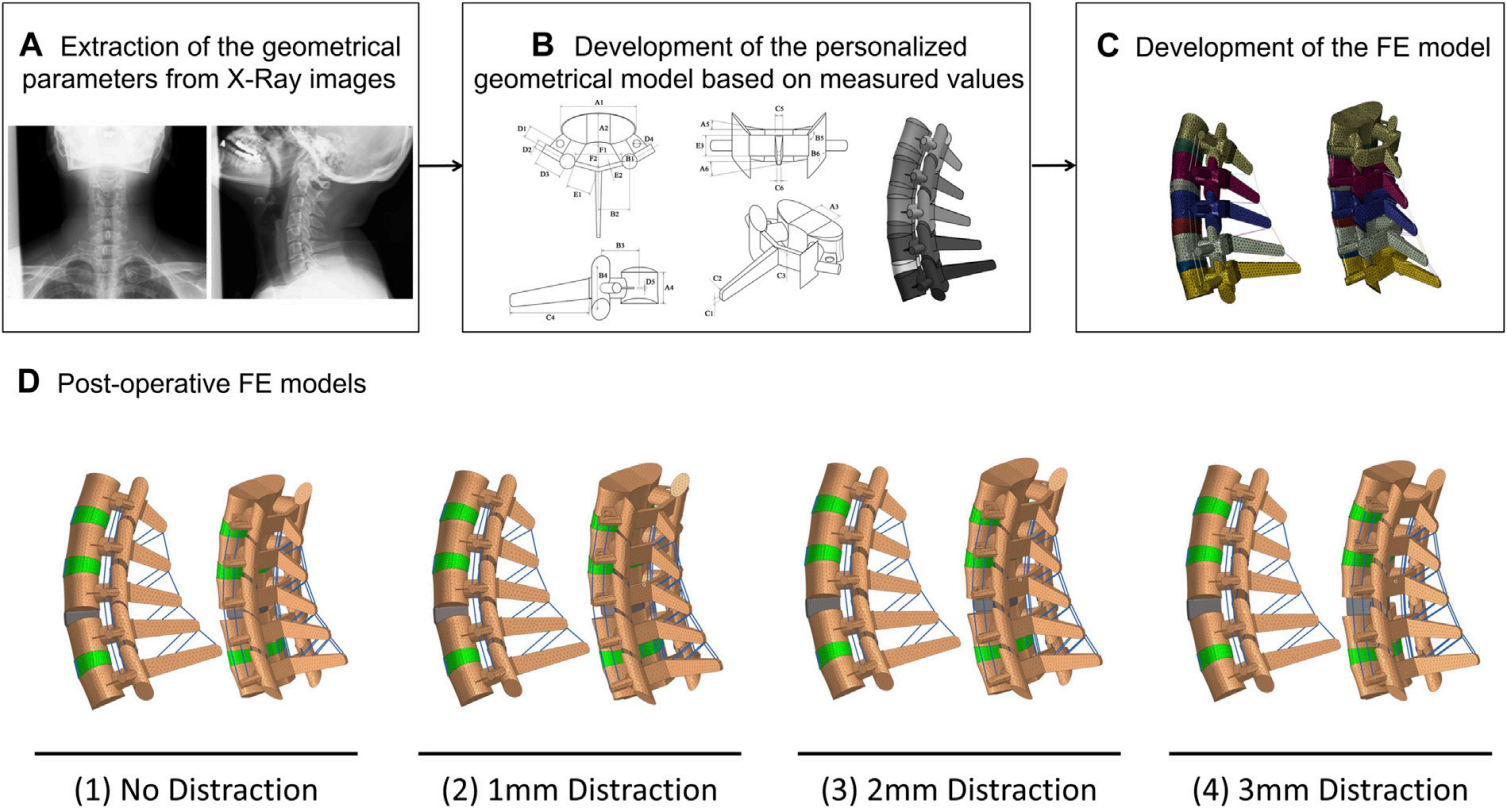
The procedure of personalized poroelastic FE modeling development: **(A)** extraction of the geometrical values **(B)** Development of the geometrical model, **(C)** Development of the poroelastic FE model. **(D)** Postoperative models including the anterior cervical decompression and fusion (ACDF) for (1) No distraction, (2) 1 mm distraction, (3) 2 mm distraction, and (4) 3 mm distraction.

The parametric values to extract the geometry of the lower-cervical spine were measured from the Anterior-Posterior (AP) and the Lateral X-ray images of 20 patients (aged 57.95 ± 8.51 years, 13 females and 7 males). The measurement procedures were performed by two different individuals, who were previously trained under the supervision of a spine surgeon, to warrant the reliability of achieved data. The pre-operative (Pre-op) geometries of the lower-cervical spine were generated based on our developed automatic algorithm (Figures 1A–C) (Nikkhoo et al., 2019; Nikkhoo et al., 2021) for all 20 patients. The X-ray images were collected from a retrospective case cohort study, in which the patients underwent one-level primary ACDF at Chang Gung Memorial Hospital from 2010 to 2017. The chosen patients exhibited symptoms of radiculopathy or myelopathy resulting from cervical degenerative pathologies, and it is worth noting that none of them had a history of osteoporosis or previous spinal surgery. Signed informed consent was acquired from all patients prior to their enrolment in the relevant clinical protocols and this study was approved by the Institute Review Board (Approval IRB No. 202200412B0) of Chang Gung Memorial Hospital.

The personalized FE models were successively developed for those assembled geometries including the typical lower cervical vertebrae from C3 to C7, four IVDs, four pairs of EPs, four pairs of facet joints (FJs), and the attached ligaments (i.e., anterior longitudinal ligament (ALL), posterior longitudinal ligament (PLL), ligamentum flavum (LF), capsular ligament (CL), interspinous ligament (ISL) and Supraspinous ligament (SSL)). To better simulate the structural nature of the IVDs, a complex material was considered including the central nucleus pulposus (NP) and surrounding annulus fibrosus (AF) regions which were reinforced by collagen fibers. In order to account for the influence of collagen fibers, we incorporated rebar elements into the ground substance matrix in six layers, arranged in an alternating crisscross pattern with a 25-degree orientation (Kumaresan et al., 1999). Porohyperelastic theory was adopted for the simulation of the nonlinear time-dependent response of IVDs (Schmidt et al., 2007a) (Table 1). Besides, the vertebral bodies and EPs were assumed based on poroelastic theory (Table 1). The permeability characteristics were calculated based on the void ratio variations during the loading/unloading scenarios (Argoubi and Shirazi-Adl, 1996; Ferguson et al., 2004; Hussain et al., 2010; Khalaf and Nikkhoo, 2022). The swelling phenomenon was mimicked using a constant boundary pore pressure technique (Galbusera et al., 2011; Nikkhoo et al., 2015), which was enforced on the external surfaces of IVDs.

**TABLE 1:** Material properties of different components of the personalized finite element model.

Component		Mechanical properties					References	
Cortical Bone		E_xx_ = 11,300 MPa, G_xy_ = 3,800 MPa, υ_xy_ = 0.484					Schmidt et al. (2006); Schmidt et al. (2007b); Schmidt et al. (2010); Toosizadeh and Haghpanahi (2011)	
		E_yy_ = 11,300 MPa, G_yz_ = 5,400 MPa, υ_yz_ = 0.203						
		E_zz_ = 22,000 MPa, G_xz_ = 5,400 MPa, υ_xz_ = 0.203						
Cancellous Bone		E_xx_ = 140 MPa, G_xy_ = 48.3 MPa, υ_xy_ = 0.45 *k*_*0*_ = 1 × 10^−20^ (m^4^/Ns), e = 0.02					Schmidt et al. (2006); Schmidt et al. (2007b); Schmidt et al. (2010); Toosizadeh and Haghpanahi (2011)	
		E_yy_ = 140 MPa, G_yz_ = 48.3 MPa, υ_yz_ = 0.315						
		E_zz_ = 200 MPa, G_xz_ = 48.3 MPa, υ_xz_ = 0.315 *k*_*0*_ = 1 × 10^−13^ (m^4^/Ns), e = 0.4						
Endplate		E = 5 MPa, ν = 0.4, *k*_*0*_ = 4 × 10^−15^ (m^4^/Ns), e = 4					Schmidt et al. (2006); Hussain et al. (2010); Toosizadeh and Haghpanahi (2011)	
Annulus Fibrosus Ground		Poro-Hyperelastic (Mooney-Rivilin)					Schmidt et al. (2006); Hussain et al. (2010); Toosizadeh and Haghpanahi (2011)	
		C1 = 0.56, C2 = 0.14, υ = 0.45, *k*_*0*_ = 1.82 × 10^−16^ (m^4^/Ns), e = 2.45						
Nucleus Pulposus		Poro-Hyperelastic (Mooney-Rivilin)					Schmidt et al. (2006); Hussain et al. (2010); Toosizadeh and Haghpanahi (2011)	
		C1 = 0.12, C2 = 0.09, υ = 0.4999, *k*_*0*_ = 1.82 × 10^−16^ (m^4^/Ns), e = 5.67						
Disc Fibers		Rebar elements, E = 500 MPa, υ = 0.3					Kumaresan et al. (1999)	
Cervical Interbody Cage		E = 3,500 MPa, ν = 0.3					Nikkhoo et al. (2020); Choi et al. (2021)	

Ligaments		Nonlinear Tension-only Truss					Kumaresan et al. (1999); Yoganandan et al. (2000); Wheeldon et al. (2008)	
		Cross-section Area of the Ligaments (mm^2^)						
Level		ALL	PLL	LF	CL	ISL	SSL	
C3-C5		11.1	11.3	46.0	42.2	13.0	9.0	
C5-C7		12.1	14.7	48.9	49.5	13.4	9.0	

*ALL, anterior longitudinal ligament; PLL, posterior longitudinal ligament; LF, ligamentum flavum; CL, capsular ligament; ISL, interspinous ligament; SSL, supraspinous ligament.

Nonlinear truss elements were used to simulate the response of ALL, PLL, LF, CL, ISL, and SSL ligaments which were defined to be activated only in tension (Kumaresan et al., 1999; Yoganandan et al., 2000; Wheeldon et al., 2008). In this anatomy-based modeling technique, the ligaments were affixed at fixed points, with their lengths adapting to the patient-specific geometry defined for the vertebrae. To simulate the articulation of the facet joints in the FE model, we implemented a surface-to-surface contact rule for both normal and tangential directions. This approach involved soft frictionless contact, with an initial gap length of 0.3 mm to mimic the facet joint articulation (Toosizadeh and Haghpanahi, 2011). The transmitted force through the contacting surfaces was modeled using an exponential pressure-overclosure relationship (Ziv et al., 1993; Schmidt et al., 2006). Other components of the FE models were considered as linear isotropic elastic materials based on available mechanical properties from literature (Table 1) (Kumaresan et al., 1999; Schmidt et al., 2006; Schmidt et al., 2007b; Toosizadeh and Haghpanahi, 2011).

Based on the meshing sensitivity investigation, each pre-op FE model used a total of 52, 723 elements for simulations. After verification of the numerical calculations, the validity of the average achieved results from pre-op FE models were evaluated compared to available *in-vitro* experimental studies in the literature (Panjabi et al., 2001; Bell et al., 2013). For this purpose, a pure moment equal to 1 N m was applied to FE models in flexion, extension, right/left lateral bending, and right/left axial rotation and the intersegmental ROM were compared to *in-vitro* experimental data by Panjabi et al. (2001). Moreover, the calculated values of IDP in a neutral position subject to a compressive follower load equal to 100 N was compared to *in-vitro* experimental data by Bell et al. (2013) to verify the validity of the fluid-solid interaction in the FE model.

### 2.2 Investigation of the influence of over-distraction on biomechanics of cervical spine post-surgery

Toward investigating the impact of anterior cervical interbody fusion and the influence of over-distraction (i.e., using larger interbody cages) on the biomechanics of the lower cervical spine, post-operative (Post-op) FE models were reconstructed for all 20 patients. Four clinical scenarios were predicted for each patient and the relevant post-op FE models were developed including (Zagra et al., 2013) No-distraction, (Robinson, 1955), 1 mm distraction, (Jacobs et al., 2011), 2 mm distraction, and (Peng et al., 2022) 3 mm distraction (Figure 1D). To achieve this aim, we have adopted the pre-op index disc height as a key parameter for defining the interbody height in the first scenario (i.e., No-distraction). For subsequent scenarios, we have made appropriate modifications based on this initial setting. To ensure the proper heights of the implanted devices, we selected standard values from the manufacturers’ product catalogues. These changes allow us to simulate and analyze the effects of different distraction scenarios on the intervertebral disc. The pre-op FE models were modified at the C5-C6 level by removing the IVD and EPs and inserting the anterior cervical interbody cage, filled with bone graft. The parametric geometry of interbody cage was designed based on the manufacturers’ product catalogue and the height values were extracted from standard ranges. The mechanical properties of the interbody cage were considered as linear isotropic elastic from literature (Table 1) (Nikkhoo et al., 2020; Choi et al., 2021). To mimic the permanent fusion in C5-C6 level, the superior and inferior surfaces of the interbody cage were attached to the vertebral bodies by means of the tie contact algorithm. Hence, 80 post-op FE models (4 post-op scenarios for 20 patients) were developed for comparative simulations.

Following a compressive pre-loading resting period for 30 min under a constant compressive load of 46N to mimic the weight of head (Plagenhoef et al., 1983; Yoganandan et al., 2009), a cyclic compression load with an amplitude of 100 N and frequency of 0.5 Hz {i.e., in the form of F = 50 + 50 cos [π(t-1)]}, was applied to the post-op FE models. This cyclic axial compressive loading was simulated based on the follower load methodology using connector elements (Patwardhan et al., 1999; Shirazi-Adl and Parnianpour, 2000; Dreischarf et al., 2014) for 11,000 cycles (Motiwale et al., 2016; Komeili et al., 2021). Rotational movements (i.e., flexion, extension, right/left lateral bending, and right/left axial rotation) were superimposed using 1 N m moment before and after cyclic loading. Those rotational moments were separately applied to the centroid of the superior surface of C3, and Dirichlet boundary conditions were considered at the inferior surface of C7. In each simulation, only one motion was evaluated for a better comprehensive comparative study and to avoid the computational errors regarding the loading combinations. Biomechanical responses, including intersegmental ROMs, IDP, FJF, IVD height loss, IVD fluid loss, maximum stress in the AF, and maximum collagen fiber strain, were analyzed before and after cyclic loading under the same loading and boundary conditions. For the robust comparative investigation based on within-subject differences, we utilized the non-parametric Friedman test followed by Nemenyi *post hoc* tests to assess the statistical significance of the results. We considered *p*-values less than 0.05 as significant for this non-parametric statistical comparative test.

## 3 Results

**FIGURE 2:**
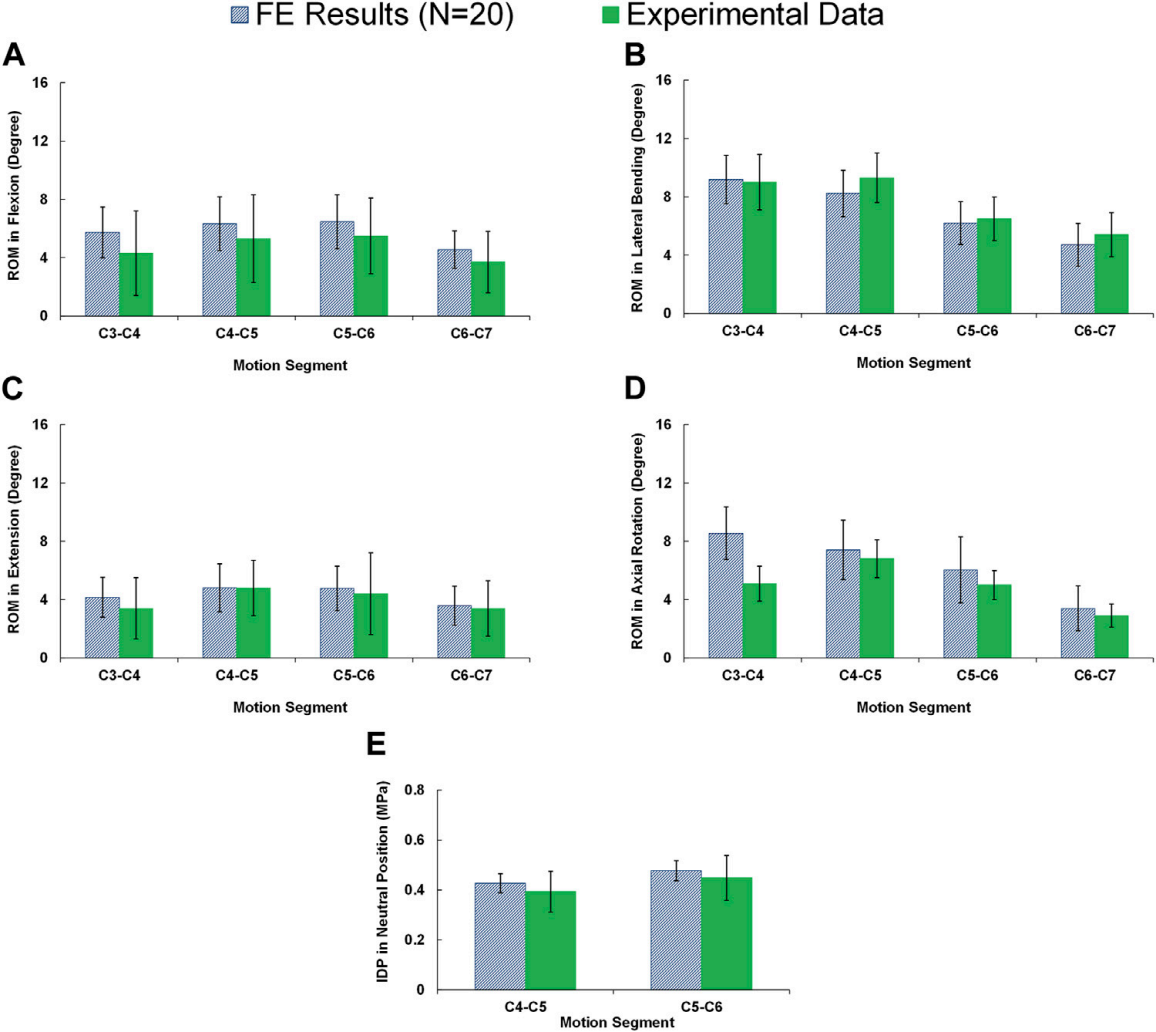
Comparison of the intersegmental rang of motions (ROMs) for pre-op FE models (N = 20) compared to *in-vitro* experiments (Panjabi et al., 2001) in **(A)** flexion, **(B)** extension, **(C)** lateral bending, and **(D)** axial rotation. **(E)** Comparison of the interadiscal pressure (IDP) for pre-op FE models (N = 20) in neutral position subject to 100N compressive follower load compared to *in-vitro* experiments (Bell et al., 2013).

All 20 pre-op lower-cervical geometries were effectively reconstructed using the developed personalized modeling technique and the accuracy of all the FE models were confirmed by assessing the mesh independency tests. The average estimated ROMs for the whole lower-cervical spine were 23.09 (±6.70), 17.31 (±5.89), 28.31 (±6.18), and 25.39 (±7.64) degrees, for flexion, extension, lateral bending, and axial rotation, respectively. These average calculated intersegmental ROMs were within the range compared to the *in-vitro* data from Panjabi et al. (Panjabi et al., 2001) (Figures 2A–D). In addition, the average calculated IDP values in the neutral position for C4-C5 and C5-C6 subject to compressive force (i.e., 100 N) were 0.427 (±0.038) and 0.477 (±0.040) MPa, respectively, which were found to be well within the reported experimental data by Bell et al. (2013) (Figure 2E).

**FIGURE 3:**
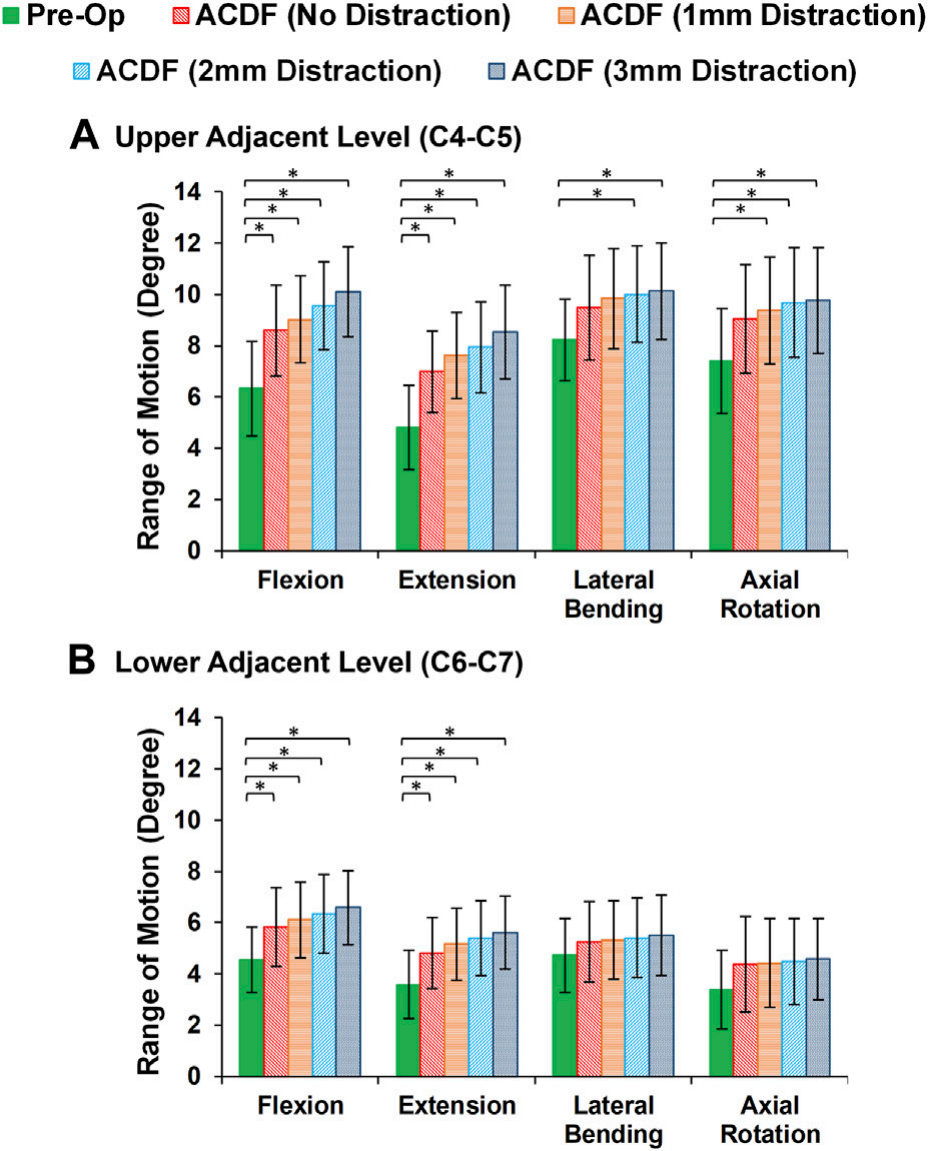
Comparison of the intersegmental rang of motions (ROMs) for lower cervical FE models at **(A)** upper adjacent level (C4–C5), and **(B)** lower adjacent level (C6–C7) for different movements.

**FIGURE 4:**
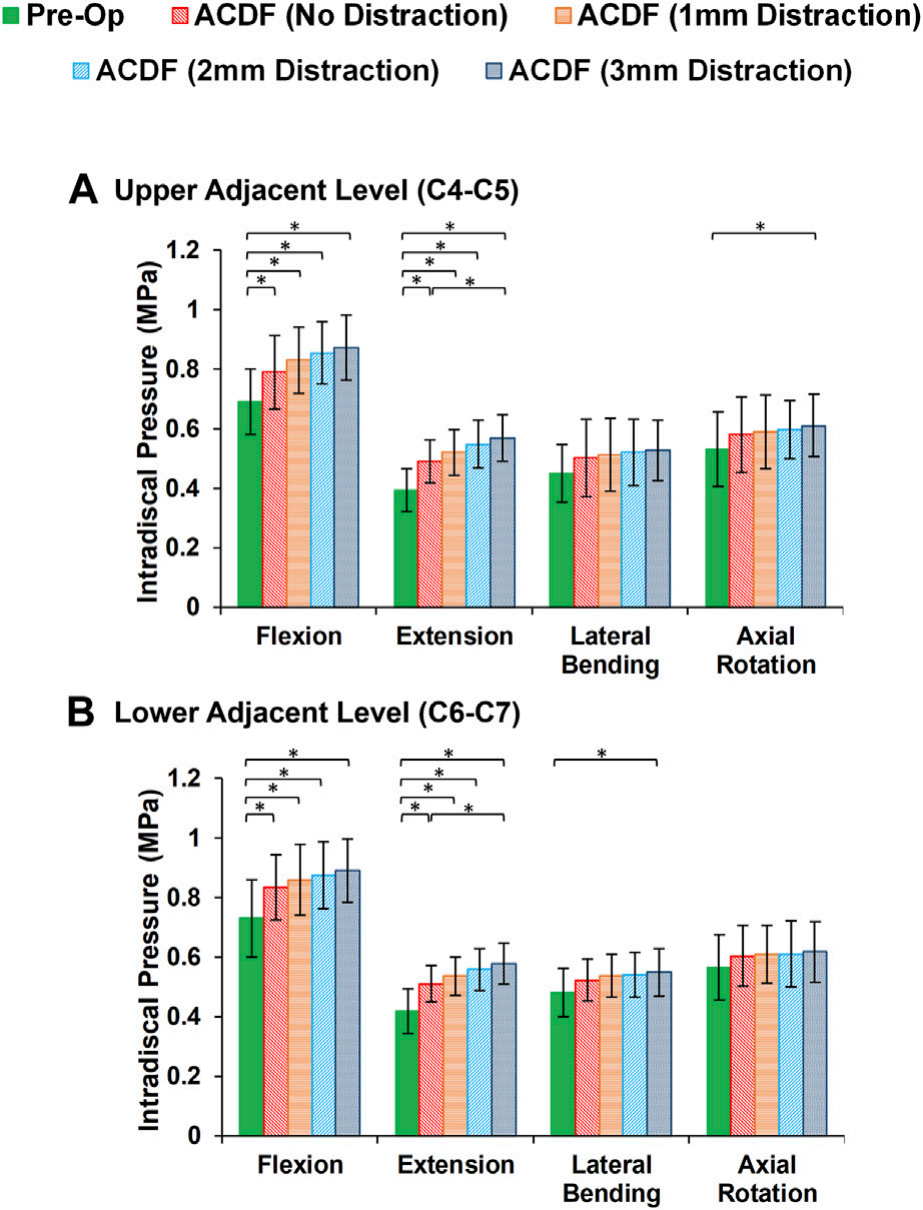
Comparison of the intradiscal pressure (IDP) values for lower cervical FE models at **(A)** upper adjacent level (C4–C5), and **(B)** lower adjacent level (C6–C7) for different movements.

**FIGURE 5:**
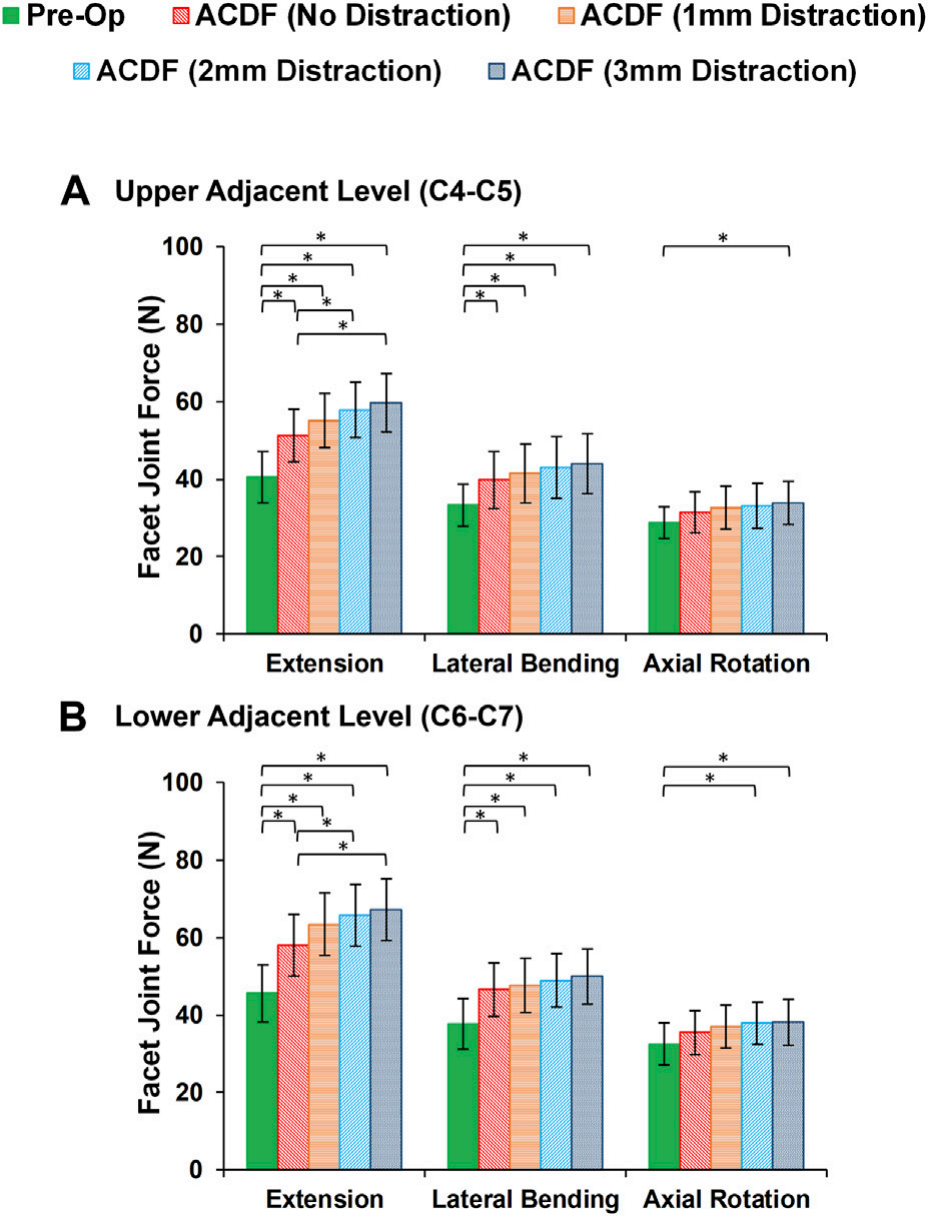
Comparison of the facet joint force (FJF) values for lower cervical FE models at **(A)** upper adjacent level (C4–C5), and **(B)** lower adjacent level (C6–C7) for different movements.

In static loading simulations, the ROMs at the upper and lower adjacent levels (i.e., C4-C5 and C6-C7 segments) significantly increased for ACDF models with no distraction during sagittal plane movement (Figure 3). These variations were 35.74% and 27.69% in flexion and 45.15% and 34.47% in extension for C4-C5 and C6-C7, respectively (Figure 3). However, only distraction groups showed significantly higher ROMs in lateral bending and axial rotation in upper adjacent level. In addition, no statistical differences were calculated between different distraction groups (Figure 3). Similar trends were calculated for IDP value variations for flexion and extension, however, significant differences between no-distraction and over-distraction groups were observed in extension (Figure 4). Higher FJF values were observed in adjacent levels for ACDF models, and over-distraction groups (i.e., distraction height≥ 2 mm) induced significantly higher FJF in both upper and lower adjacent levels (Figure 5). FJF values in flexion were not reported as the they remained unloaded, because the opposing facet surfaces displaced apart from each other during this movement (Schmidt et al., 2008).

**FIGURE 6:**
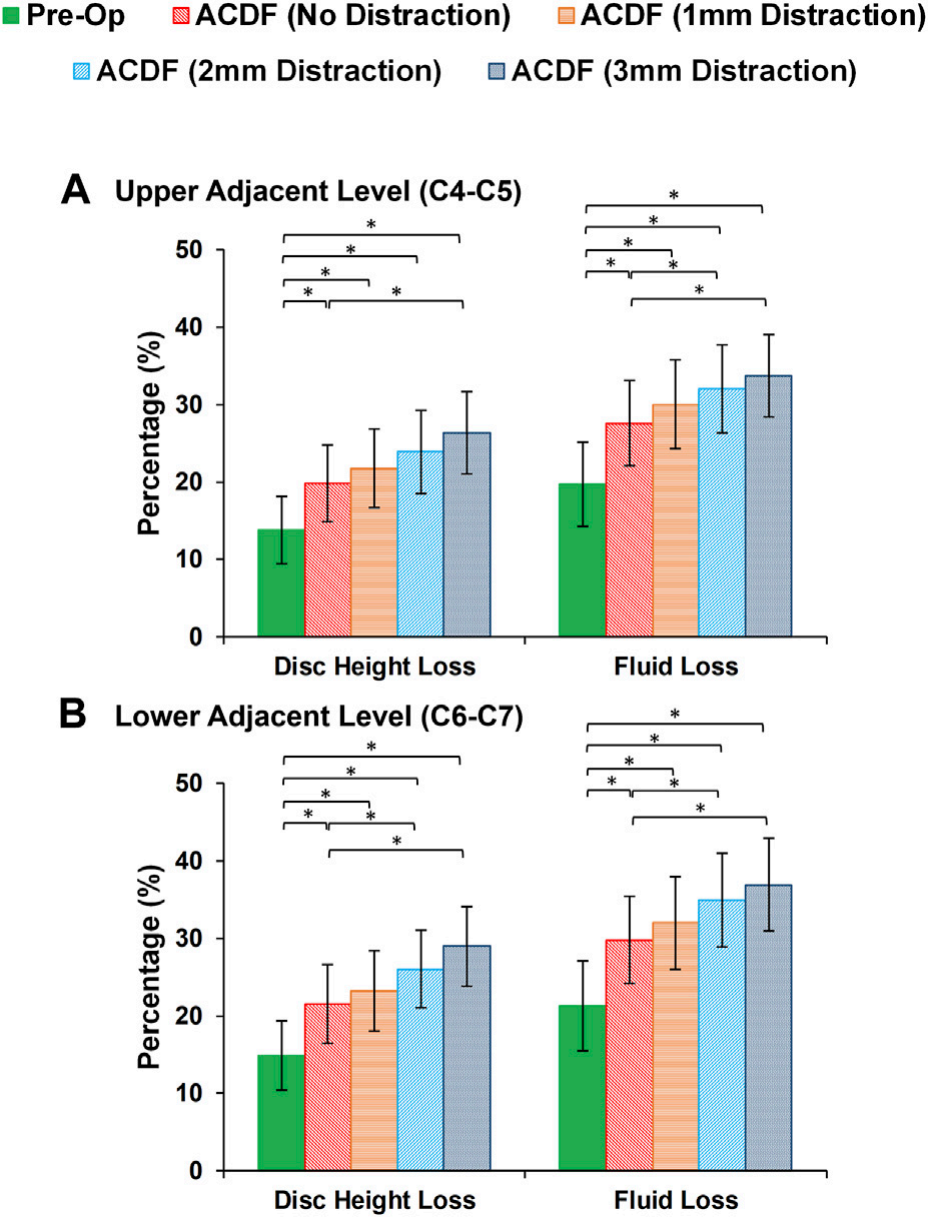
Comparison of the intervertebral disc height loss and fluid loss for pre-op (intact) and post-op lower cervical FE models at the **(A)** upper adjacent level (C4-C5) and **(B)** lower adjacent level (C6-C7).

At the end of cyclic loading simulations, the pre-op FE models averagely showed 13.79 (±4.32) %, 14.31 (±4.36) % and 14.88 (±4.47) % reduction of disc height at the C4-C5, C5-C6, and C6-C7, respectively. Correspondingly, the average fluid loss for pre-op models were 19.69 (±5.46) %, 20.57 (±5.61) and 21.26 (±5.80) % at the C4-C5, C5-C6, and C6-C7, respectively. For the post-op models, both of these parameters were significantly increased (Figure 6). Over-distraction during ACDF (i.e., distraction height≥ 2 mm) showed significant alteration in both IVD height loss and fluid loss (Figure 6).

**FIGURE 7:**
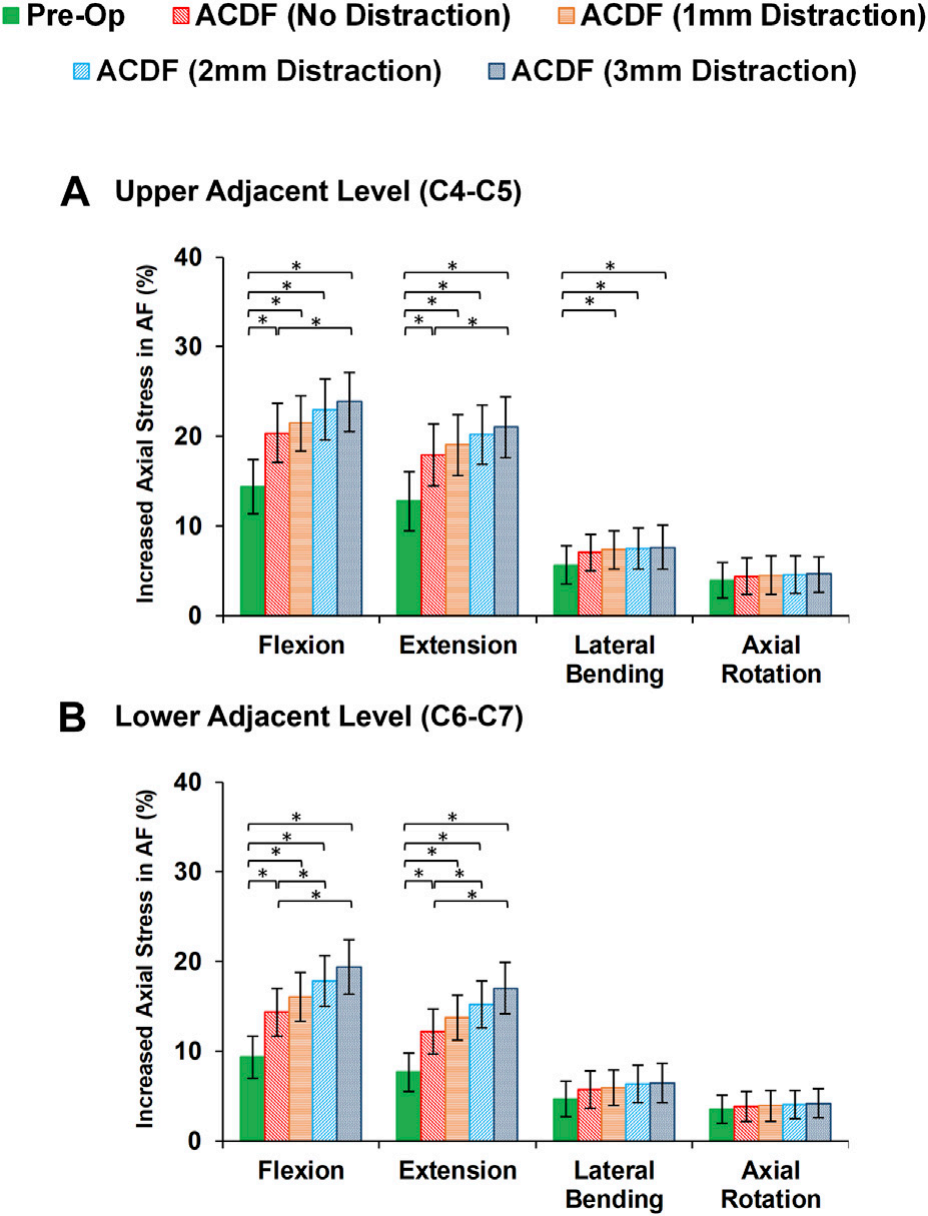
Comparison of the increased axial stress in AF for pre-op (intact) and post-op lower cervical FE models at the **(A)** upper adjacent level (C4-C5) and **(B)** lower adjacent level (C6-C7).

**FIGURE 8:**
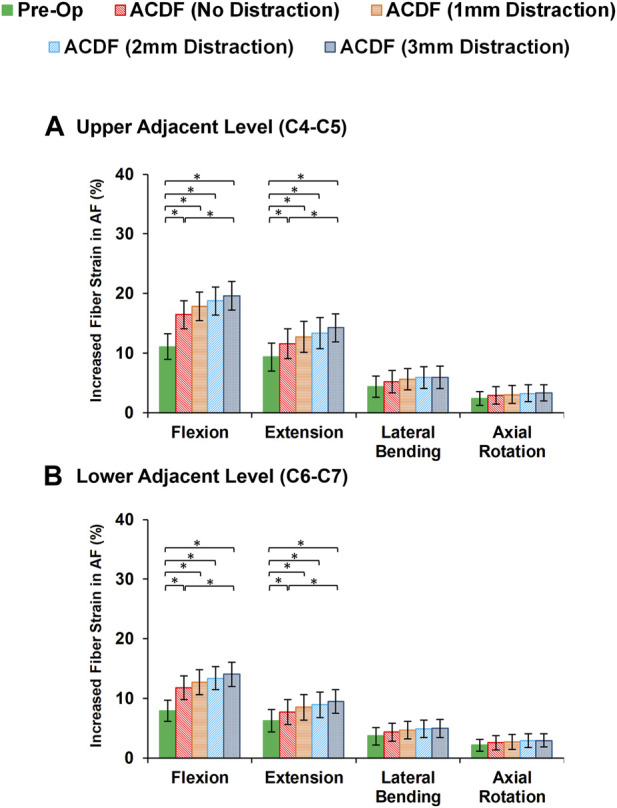
Comparison of the increased fiber strain in AF for pre-op (intact) and post-op lower cervical FE models at the **(A)** upper adjacent level (C4-C5) and **(B)** lower adjacent level (C6-C7).

Variations of AF axial stress and fiber strain averagely showed similar patterns, in which significantly higher values were calculated for ACDF models and these values further increased for over-distraction groups in sagittal plane movements (Figure 7). Except for the significantly increased axial stress in the upper adjacent level, no other significant differences were detected in comparative tests between the calculated values of AF axial stress and fiber strain at the adjacent levels in different FE model groups during lateral bending and axial rotations (Figure 8).

## 4 Discussion

The ACDF surgical technique continues to be widely used as the gold standard treatment for cervical degenerative IVD diseases. The use of PEEK interbody cage has since evolved to become prevalent in last two decades which tolerates good load sharing and minimizes the stress-shielding effect (Song and Choi, 2014; Jain et al., 2016). Nonetheless, it is still a main challenge for surgeons to select the most proper interbody cage height for different patients (Kwon et al., 2005; Chong et al., 2015). The decision-making regarding the interbody cage height may be planned based on the surgeon’s experience, patient’s demographics, and pathological conditions. Distraction of the IVD space is one of the main steps of ACDF surgical technique which allows better visualization during the removal of the IVD and exposing the blood-rich cancellous bone underneath (Robinson, 1955; Olsewski et al., 1994). Some surgeons prefer over-distraction and implanting the interbody cages with larger height size. Currently, a universally accepted consensus or standardized definition regarding the specific threshold for distraction displacement that categorizes cervical spine surgery as either “over-distraction” or “normal distraction” in the context of ACDF does not exist (Kirzner et al., 2018; Hah et al., 2020; Lawless et al., 2022). The classification of over-distraction largely depends on individual surgeons, specific medical institutions, and the relevant clinical guidelines they follow, which are influenced by their collective experience. Due to this lack of uniformity, the identification of what constitutes over-distraction remains subjective and variable across different healthcare settings and practitioners. Over-distraction with excessive force during the surgery may lead to an increased risk of injuries to the FJs at the index level (Olsewski et al., 1994). In addition, variation of the interbody cage height may alter the biomechanical response of adjacent levels post-surgery (Igarashi et al., 2019; Huang et al., 2021). In our study, we endeavoured to establish a definition of distraction in ACDF surgery by quantifying the discrepancy between postoperative and preoperative index disc heights. After analysing the existing data in the literature and drawing from the combined experience of the participating surgeons, we determined that a difference equal to or exceeding 2 mm could be regarded as indicative of “over-distraction”. A comparative investigation on assessing the response of the lower cervical spine post-ACDF with different distraction conditions is hence critical for informed surgical and treatment planning.

To achieve this purpose, we used a validated personalized poroelastic FE modelling approach and comprehensively performed the simulations for 20 patients (A total of 100 FE models including a 20 pre-op and 80 post-op FE models) subjected to static and cyclic loading conditions. The primary objective of static analyses is to ascertain the deformation and stress distribution in the cervical spine under conditions where the loading remains constant during certain postures or movements that change slowly over time. This type of simulation is widely used in the literature (Schmidt et al., 2013; Kim et al., 2018) and allows for comparative investigations between pre-op and post-op conditions. However, it is important to note that biological tissues, especially the IVD, which significantly contributes to the biomechanical response of the cervical spine, exhibit time-dependent behavior that varies during daily loading conditions (Nikkhoo et al., 2013). To gain a deeper understanding of the cervical spine’s biomechanical response after surgical manipulations, incorporating cyclic loading scenarios can prove beneficial. Cyclic loading analyses are crucial in exploring how the cervical spine responds to repeated loading and unloading cycles, reflecting real-world scenarios. Therefore, one of the significant contributions of this study was to provide simulation results for both static and cyclic loading conditions, facilitating a more realistic and comprehensive comparative analysis. This approach acknowledges the time-dependent behavior of biological tissues and offers a more comprehensive assessment of the cervical spine’s response to various loading conditions, thus advancing the understanding of its biomechanical behavior post-surgery.

The accuracy of the FE calculations were confirmed based on mesh sensitivity analyses and the estimated results obtained from 20 personalized FE models fell within the overall ranges when compared to the available data from the existing literature (Panjabi et al., 2001; Bell et al., 2013). This confirmation ensures the reliability of our FE simulations and strengthens the credibility of the study’s findings. Only the average calculated ROM in axial rotation at the upper level (i.e., C3-C4) was significantly higher than the reported data by Panjabi et al. (2001) (Figure 2). This issue may refer to the simplified geometry of the FJ and neglecting the upper cervical region, nevertheless, it is common in FE model studies not to completely match with *in-vitro* data for all details in different movements. Incidentally, an important strength point of this study was repeating the FE simulations for 20 different patients to include the effect of individual’s anatomical parameters (such as vertebral dimensions, IVD heights, and cervical lordosis angles). During the validation phase, the results of intersegmental ROMs and IDP values exhibited significant variations. This emphasizes the substantial impact of geometrical parameters on the kinematics and kinetics of the cervical spine. The observed large variations underscore the importance of considering precise geometrical factors in accurately predicting the biomechanical behavior of the cervical spine. Most of related FE models in literature performed the simulations based on only one particular geometry (Kim et al., 2018) which may limit the achieved results by neglecting the effect of anatomical parameters (Laville et al., 2009; Nikkhoo et al., 2019). On the other hand, no statistical comparative test could be performed based on the results from only one geometry to evaluate if the calculated variations between pre-op and post-op results were statistically significant or not. Therefore, this study performed comprehensive FE simulations for 20 patients and non-parametric statistical comparative tests were utilized to evaluate the effect of selecting larger-sized interbody cages during ACDF under both static and cyclic loading.

In this study, we developed a personalized FE modeling technique based on simple Lateral and AP X-Ray images in the upright posture. X-Ray imaging was chosen due to its commonality, simplicity, and widespread availability, making it a cost-effective alternative to MRI and CT scans. Despite the advantages of CT scanning, issues such as radiation exposure (Lin, 2010) and the supine posture during imaging, which does not reflect the cervical spine’s alignment during normal daily activities, limit its applicability (Hasegawa et al., 2018). One crucial aspect of our work was the focus on clinical applicability and functionality, which we extensively discussed in our previous research (Nikkhoo et al., 2019). The developed interface allowed clinical staff with no prior FEM experience to perform parameter extraction. After training, the measurement procedure was carried out independently by two different individuals, and the process was repeated three times, demonstrating good intra- and interobserver reliability. The geometrically-personalized FE model we created can be automatically generated by inputting parameter values in clinics without the need for programming or FE modeling knowledge. The main advantage of the parametric modeling approach used in this study is its ability to significantly simplify the geometrical personalization process, resulting in reduced calculation time. Although some geometrical details were sacrificed using parametric modeling, it allows for easy modification of the geometry to mimic various spinal pathologies or treatment manipulations.

To enhance the estimates for both short-term and long-term outcome of ACDF surgery, the nonlinear poroelastic theory was utilized in this study. Incorporating the time-dependent interactions of interstitial water in the saturated solid matrices of IVDs, EPs, and vertebral bodies could yield more realistic predictions for conducting comparative analyses between pre-op and post-op models. By accounting for the dynamic behavior of interstitial water, we can better capture the biomechanical response and changes in these structures over time, thereby enhancing the accuracy and reliability of the comparative analyses between different surgical scenarios. In most of previous FE studies in cervical spine biomechanics, only static loadings (i.e., static rotational movements of cervical spine in different anatomical planes) were applied for comparative simulations (Kim et al., 2018; Chen et al., 2020; Hua et al., 2020). In this study, the pre-op and post-op models were evaluated subject to static rotational movements and then a repetitive compressive cyclic loading scenario [11,000 cycles with an amplitude of 100 N and frequency of 0.5 Hz (Motiwale et al., 2016; Komeili et al., 2021)] were applied. Further, the static rotational movements were repeated at the end of cyclic loading for comparative investigations. Variations in IVD height loss, fluid loss, AF stress, and fiber strain in adjacent levels (i.e., C4-C5 and C6-C7) were evaluated which could be defined as the mechanical indexes for increasing the risk factor of ASD. Although ASD is a long-term phenomenon that is perhaps initiated and developed 12–60 months post-surgery, this long-term procedure cannot be realistically simulated due to computational complexity. The IVD height loss and fluid loss are two quantitative clinical indicators to predict the risk of ASD (Urban and Roberts, 2003; Suzuki et al., 2017). Furthermore, comparisons of the increased stress and strain in adjacent IVDs before and after cyclic loading possibly will represent another indicator for the accumulative risk of adjacent IVD degeneration. Therefore, the variations of the above-mentioned parameters were deliberated in this study for the prediction of the risk factors for the initiation of ASD.

The findings based on the comparison of the pre-op and post-op FE simulations in static loading revealed that the calculated ROMs at adjacent levels significantly increased in ACDF models for sagittal plane movement. The ROMs for the post-op models with larger interbody cages significantly increased for upper adjacent levels in lateral bending and axial rotation, as well. Similar trends were observed in IDP and FJF values but significant differences were, moreover, detected for over-distraction in extension movement (Figures 4, 5). ACDF at instrumented level (i.e., C5-C6) prevents movement between the fused vertebrae and potentially the adjacent levels compensate for the motions which may technically result in higher experienced ROM, IDP, and FJF. Excessive over-distraction has a notable effect on the alignment of the lower cervical spine in adjacent levels, resulting in a significant increase in tension within the adjacent FJs. These findings indicate that using a larger interbody cage in cases of over-distraction might contribute to an elevated risk of facet joint degeneration and failure in the adjacent level (Kirzner et al., 2018). The altered alignment caused by over-distraction places additional stress on the adjacent facet joints, potentially leading to accelerated wear and tear, degeneration, or even mechanical failure over time (Hah et al., 2020; Lawless et al., 2022). These implications highlight the importance of carefully assessing and selecting the appropriate interbody cage size during ACDF procedures to avoid potential complications and promote long-term spinal health and stability. In a previous study exploring the effects of artificial disc arthroplasty height on cervical biomechanics (Yuan et al., 2018), it was demonstrated that implants with an over-distraction index height (≥2 mm greater than the index disc height) significantly elevated the adjacent IDP, FJF, and experienced stress when compared to the index disc height.

Evaluating the response of FE models subjected to cyclic loading can reveal an enriched possibility to include the time-dependent characterization of cervical spine which can be generalized for the prediction of long-term outcomes. Disc height and interstitial water were uniformly reduced during cyclic loading in different segments for intact pre-op FE models. The ACDF modifications in post-op FE models altered the IVD height loss and fluid loss that can be considered key indicators of IVD denaturation and degeneration initiation. Increasing the rigidity of the fused segment modifies load sharing pattern through the adjacent levels, which leads to accumulative IVD height loss and fluid loss. This study showed that significant differences were observed between calculated disc height loss and fluid loss of ACDF with no distraction and over-distraction groups (distraction height≥ 2 mm). Similar comparative trends were observed for both experiences of stress in AF region and collagen fiber strain. Greater interstitial water loss may possibly reduce the contribution of the fluid phase to the overall resistance of the disc structure. Therefore, the calculated values of axial stress in AF region and the collagen fiber strain might respectively increase. The findings mentioned above suggest that using an inappropriate interbody cage height may induce an abnormal biomechanical response in adjacent levels, potentially leading to the development of ASD over the long term. An insightful clinical study highlighted a statistically significant correlation between increased facet joint distraction and worsened neck disability index (NDI) and visual analogue scale (VAS) pain scores (Kirzner et al., 2018). The analysis revealed that an optimal amount of FJ distraction was 2 mm or less, whereas patients distracted by 3 mm or more exhibited notably worse VAS pain and NDI scores (Kirzner et al., 2018). Another comprehensive clinical investigation shed light on the prevalence of cervical axial symptoms (AS) following ACDF surgery (Bai et al., 2015). The occurrence of AS was attributed to changes in the curvature of the cervical fusion segment after surgery and over-distraction of the surgical segment. Reducing AS after surgery was found to be linked to moderate distraction of the intervertebral space and the use of appropriately sized interbody cages (Bai et al., 2015). It is imperative in ACDF surgery to ensure its efficacy, prevent intervertebral collapse, kyphosis of fused segments, and mitigate the risk of AS by exercising caution to avoid over-distraction of the surgical segment. Taking these factors into account during surgical planning can lead to improved patient outcomes and a more successful recovery process (Bai et al., 2015; Lawless et al., 2022; Tsalimas et al., 2022).

The limitations of this comprehensive FE investigation should be reflected as well. The first aspect pertains to the development of the FE models, which were constructed based on simple symmetric shapes (such as circles, rectangles, and ellipses) using X-Ray images, rather than relying on detailed geometry derived from CT-scan images. On the other hand, our previous comparative analyses, where both parametric and geometrically-accurate models with identical geometry were employed, revealed similar trends in the global response (e.g., ROM, IDP, fiber strain). This observation confirms the effectiveness of this modeling approach (Nikkhoo et al., 2014; Nikkhoo et al., 2019). Moreover, the simplified parametric technique adopted in this study offers additional benefits in terms of reduced time and computational cost. Furthermore, it facilitates easy updates with patient-specific data, making it highly suitable for clinical applications. The advantages of this approach justified the simplifications made and enhanced its clinical applicability significantly. We included the effect of anatomical parameters based on our previously developed personalized FE modelling technique, however, the adopted mechanical properties in those different models were not patient-specific. Due to the limitations of X-Ray images, it was not feasible to differentiate and derive personalized material properties for biological tissues in our study, it was an unavoidable limitation and we used similar mechanical properties for all FE models as described in Table 1. This constraint could be tolerated as the main objective of this FE investigation was to comparatively evaluate the effect of interbody cage height and over-distraction. It is crucial to highlight that none of the selected patients in this study had a history of osteoporosis. The absence of osteoporosis among the chosen participants is a consideration that may minimize the effect of this simplification, as osteoporosis could potentially influence the biomechanical behavior and surgical outcomes. In addition, we considered the follower load technique to mimic the passive response of muscle forces in the cervical spine, however, the effect of active muscle forces was neglected. This is a common simplification in FE modelling of cervical spine and may have a minor effect on achieved results from this study as the pre-op and post-op FE models were compared in the same loading conditions. Furthermore, to provide a better comparative study, it was assumed that interbody cages were attached to the vertebral bodies by means of the tie contact algorithm to mimic a perfect fusion. However, it possibly will not happen for all patients and interbody cage migration or subsidence after ACDF is one of the major concerns for spinal surgeons which was not investigated in this study.

In conclusion, the current study proposed a comprehensive FE investigation using personalized modeling technique to comparatively evaluate the effect of implanting larger-sized interbody cages during ACDF surgery. The model predictions reveal greater ROM, higher values of IDP, FJF, stress and strain in the AF region, and increased disc height and fluid loss at the adjacent levels for ACDF, as compared with intact pre-op models, which may indicate as risk factor for ASD. Furthermore, over-distraction using larger sized interbody cages significantly increased the IDP, FJF, disc height loss, fluid loss, stress and strain in the AF region in adjacent segments, as well. Consequently, this study concluded that the utilization of larger-sized interbody cages (with a height of ≥2 mm relative to the index disc height) could lead to significant variations in biomechanical responses in adjacent levels. This underscores the critical importance of carefully selecting the appropriate height of the interbody cage in ACDF surgery. Proper cage height selection is essential to maintain optimal biomechanical stability and reduce the risk of potential complications in the adjacent levels following the surgical intervention.

## Data Availability

The raw data supporting the conclusion of this article will be made available by the authors upon request.
